# Metastasis-associated DNA methylation alterations persist after accounting for immune and stromal cell heterogeneity in primary colorectal tumors

**DOI:** 10.21203/rs.3.rs-9657747/v1

**Published:** 2026-07-14

**Authors:** Alos B. Diallo, Sabin D. Hart, John P. Zavras, Scott M. Palisoul, Fred W. Kolling, Louis J. Vaickus, Jiaoyuan E. Sun, Lucas A. Salas, Brock C. Christensen, Joshua J. Levy

**Affiliations:** Dartmouth College; Dartmouth College; Dartmouth College; Dartmouth–Hitchcock Medical Center; Dartmouth Cancer Center; Dartmouth–Hitchcock Medical Center; Dartmouth–Hitchcock Medical Center; Dartmouth College; Dartmouth College; Cedars-Sinai Medical Center

**Keywords:** epigenetics, DNA Methylation, colorectal cancer, tumor microenvironment, epigenomewide association study

## Abstract

**Background:**

Metastatic colorectal cancer (CRC) remains a major cause of cancer mortality, yet how epigenetic states within the tumor microenvironment (TME) relate to metastatic progression has not been fully characterized. Although aberrant DNA methylation has been implicated in cancer progression, many studies do not account for immune and stromal cell composition, which can confound bulk methylation analyses. We sought to define DNA methylation patterns in primary colorectal tumors associated with lymph node and distant metastasis, while accounting for tumor microenvironment cellular composition.

**Methods:**

We analyzed DNA methylation patterns from 57 patients with stage pT3 colorectal adenocarcinoma, comparing tumors with and without concurrent metastasis. Methylation cytometry deconvolution was performed to estimate immune, stromal, and tumor cell fractions. Differentially methylated positions associated with metastatic status were identified using covariate-adjusted epigenome-wide association analyses, with genomic context enrichment to assess regional methylation patterns. Genomic context enrichment and gene annotations were used to assess biological relevance. Methylation-expression correlations were assessed in an independent TCGA-COAD cohort to evaluate whether discovery loci correspond to differences in gene expression.

**Results:**

Metastatic status was not associated with large-scale differences in inferred cellular composition. Epigenome-wide analyses identified hypomethylated loci mapped to genes involved in Wnt/β-catenin and PI3K/Akt signaling, epithelial–mesenchymal transition, and immune modulation, whereas hypermethylated loci tracked to genes related to adhesion, Wnt signaling, cytoskeletal organization, and interferon signaling. Genomic context enrichment revealed CpG island enrichment among hypermethylated DMPs in the distant metastasis contrast. Among metastasis-associated CpGs, 23 showed significant negative correlations between methylation and gene expression in TCGA-COAD (FDR < 0.05), predominantly at promoter-proximal CpG islands, supporting a relationship between promoter methylation and reduced gene expression. Two candidate CpGs showed directional concordance with significance in matched TCGA contrasts.

**Conclusions:**

We identify distinct epigenetic alterations associated with metastatic progression in colorectal cancer that are largely independent of bulk immune and stromal composition. Integration of methylation-expression data supports a role for promoter hypermethylation in metastasis-associated gene silencing. These findings highlight potential methylation-based biomarkers of metastatic risk and may inform future precision oncology strategies in CRC.

## BACKGROUND

Approximately 20% of colorectal cancer (CRC) patients are diagnosed with metastatic disease, and those with distant metastases have a five-year relative survival rate of 15.6%.^1,2^ Regional lymph-node involvement, even in the absence of distant metastasis, is also a major driver of recurrence, impacting prognosis and quality of life.^3^ In contrast, there is a 91.5% 5 year survival rate and markedly reduced risk of recurrence in patients with localized CRC, underscoring the devastating impact of metastasis on long-term outcomes.^2^ CRC metastasis is often studied in the context of specific genetic mutations; however, an increasing body of work indicates that epigenetic alterations in tumor tissue and the cellular composition of the tumor microenvironment (TME) also play critical roles in disease progression.^4,5^

In colorectal cancer, aberrant DNA methylation (DNAm) patterns, including both hypermethylation and hypomethylation, are common and contribute to tumorigenesis and metastatic potential.^6,7^ Epigenetic alterations enable tumors to adapt to environmental and immune pressures, including modulation of immune cell recruitment and function within the TME.^8^ Tumor-infiltrating lymphocytes (TILs), including T-cells, can directly kill cancer cells, activate other immune cells, and shape metastatic potential.^9^ In addition, stromal cell populations also play a critical role in shaping metastatic behavior, for example, cancer-associated fibroblasts (CAFs) modulate extracellular matrix remodeling in the TME, engaging key processes associated with metastasis.^10^ Because each cell type has distinct methylation patterns, shifts in TME composition can confound bulk methylation measurements, necessitating cell-type deconvolution to identify tumor-intrinsic epigenetic alterations associated with metastasis.^11–13^ To address this limitation, we leveraged methylation cytometry deconvolution, implemented using two complementary DNA methylation-based methods – Hierarchical Tumor Immune Microenvironment Epigenetic Deconvolution (HiTIMED) and Epigenetic Dissection of Intra-Sample Heterogeneity (EpiDISH) – to quantify tumor, immune, and stromal cell proportions from bulk methylation data.^14–16^

Prior research has implicated aberrant DNA methylation patterns, both hypermethylation and hypomethylation, in CRC tumorigenesis and progression.^6,7^ For example, promoter hypermethylation of mismatch repair genes including *MLH1* frequently leads to microsatellite instability (MSI), while global hypomethylation of intergenic regions promotes chromosomal instability and oncogene activation.^6,7^ In the context of disease progression, several studies have shown that primary CRCs exhibit stage-linked molecular differences, including distinct methylation-defined subgroups and stage-restricted prognostic methylation signatures.^17,18^ Beyond individual loci, multi-CpG signatures measured from resected primary tumors have prognostic value for recurrence, distant metastasis, and survival beyond standard clinicopathologic factors.^19^ Additionally, methylation signatures of immune cell populations, including CD8^+^ T-cells have been leveraged to construct prognostic indicators for patients with CRC.^20^

Many prior studies of metastasis-associated DNA methylation have focused on comparisons between primary tumors and established metastatic lesions, identifying epigenetic differences at secondary sites rather than variation within primary tumors across stages of disease.^5,21,22^ Studies comparing early-stage to advanced-stage primary tumors may conflate local invasion depth with metastatic dissemination, making it difficult to isolate methylation alterations that specifically associate with lymphatic or hematogenous spread.^19,23,24^ Here, we focus on DNA methylation patterns within resected primary CRC tumors, restricted to pT3 stage (tumor-stage 3, muscularis propria invasion), to identify how epigenetic variation relates to local and distant metastatic progression (Stage II vs. III vs. IV). Prior studies do not typically account for underlying cellular composition differences between primary tumors, presenting a unique opportunity to better isolate intrinsic molecular alterations associated with metastatic progression.

We hypothesize that distinct DNA methylation patterns distinguish metastatic from non-metastatic colorectal tumors, and that accounting for cellular heterogeneity enables accurate identification of these tumor-intrinsic epigenetic alterations. We examined metastasis-associated DNA methylation changes in 57 pT3 colorectal adenocarcinomas using cell-type-aware epigenome-wide association analysis, with independent validation in the The Cancer Genome Atlas Colon Adenocarcinoma (TCGA COAD) cohort.

## METHODS

### Study Population and Sample Preparation

Our retrospective cohort consists of 57 patients (24 female, 33 male) from Dartmouth Hitchcock Medical Center (IRB STUDY02000825, approved by Dartmouth Health Institutional Review Board), previously diagnosed with colon adenocarcinoma, ranging in age from 33 to 93 years (mean = 66.3, SD = 15.3). Tumor samples were resected between 2016 and 2018, with clinical follow-up through 2023. All metastatic disease (lymph node and/or distant metastasis) was identified during initial surgical staging and represents baseline pathologic characteristics rather than incident events during follow-up. Analysis was restricted to pT3 tumors to isolate DNA methylation patterns associated with metastatic spread independent of primary tumor invasion depth. Tumor-stage 3 (pT3) tumors have penetrated through the muscularis propria into pericolonic or perirectal tissue but exhibit heterogeneous metastatic behavior, enabling comparison of non-metastatic, lymph-node–only, and distant metastatic disease at a consistent level of local invasion. Based on surgical pathology, patients were stratified into three metastatic groups: no metastasis (pT3N0M0; pathologic stage II, n = 25), lymph-node–only metastasis (pT3N+M0; pathologic stage III, n = 13), and distant metastasis (pT3N±M1; pathologic stage IV, n = 19). Tumor samples were encased in formalin-fixed paraffin-embedded (FFPE) tissue blocks. DNA was extracted from FFPE tissue sections using the QIAamp DNeasy Tissue Kit (Qiagen), and bisulfite-converted using the EZ DNA Methylation Kit, following the manufacturer’s instructions. Illumina Infinium Human Methylation EPIC arrays were used to quantify DNAm, with 32 samples run on the EPIC v1.0 platform and 25 samples on the EPIC v2.0 platform.

### Quality Control and Data Pre-processing

Pre-processing steps for the DNAm data were conducted using the QCinfo function from SeSAMe to remove poor-performing CpGs based on detection p-value > 0.05, bead count = 3, and probes with known SNPs or cross-reactivity issues.^25,26^ We then performed normalization using the Minfi preprocessNoob function.^27^ CpG data from EPIC v1 was harmonized with EPIC v2 using betasCollapseToPfx, and CpGs specific to EPIC v2 were excluded.^25^ *SeSAMe* was used to perform comprehensive probe quality masking, bleed-through correction in background subtraction, nonlinear dye bias correction, non-detection calling, and to control for bisulfite conversion based on C/T-extension probes.^25^ At individual CpG sites, DNAm is quantified as the proportion of methylated cytosines across a population of DNA molecules (beta values, ranging from 0 to 1).^28^ To improve suitability for linear modeling, beta values were converted to M-values (logit-transformed β-values) using the BetaValueToMValue function in SeSAMe.^25^ To reduce the burden of multiple comparison corrections, the top 10% of CpGs (68,331 CpG sites) with the most variable beta values across subjects were considered. Multicollinearity was assessed using the Variance Inflation Factor (VIF) from the R package regclass.^29^ Analyses were conducted in R version 4.5.1.

### Statistical Analysis

Methylation cytometry deconvolution to quantify cell types in the TME was conducted using the HiTIMED (COAD) and EpiDISH (*centEpiFibIC.m* reference) packages in R.^14,15,30^ HiTIMED was used to quantify cell type proportions for all cell types except for fibroblasts, which were quantified using EpiDISH. Because HiTIMED and EpiDISH use distinct reference libraries and deconvolution algorithms, concordance between their overlapping cell-type estimates was assessed (Supplementary Figure S3). Cell-type proportions were compared across metastatic groups using Kruskal-Wallis tests. Individual cell types as well as aggregated compartments (immune, lymphocyte, myeloid, stromal, granulocyte) were assessed. For immune cell subsets, proportions were normalized by the total immune fraction to assess relative immune composition independent of overall immune infiltration levels. Cell-type proportions estimated from our cohort were also compared against tumor-adjacent colon samples from the Cancer Genome Atlas (TCGA) COAD cohort and healthy colon samples from the Genotype-Tissue Expression (GTEx) project, which served as non-tumor reference tissues to confirm that tumor samples exhibited distinct cellular composition profiles relative to normal colon tissue (Supplementary Figure S1).^31,32^ To enable direct comparison of non-tumor compartments across tissue types, immune, epithelial, endothelial, and fibroblast proportions were renormalized after excluding the tumor fraction, ensuring that compositional differences were not driven by the displacement of non-tumor cell types by the tumor compartment. Immune cell subsets (CD8^+^ T-cells, Tregs) were expressed as fractions of total immune and did not require renormalization. Pairwise comparisons across GTEx healthy colon (n = 297), TCGA tumor-adjacent normal colon (n = 45), and discovery cohort tumors (n = 57) were performed using Wilcoxon rank-sum tests with Benjamini-Hochberg correction.

An epigenome-wide association study (EWAS) was conducted using limma linear models with empirical Bayes moderation to identify differentially methylated positions associated with metastatic status (any metastasis, lymph-node–only metastasis, or distant metastasis). Cell-type covariates were selected to adjust for major sources of cellular heterogeneity in the tumor microenvironment. Fibroblasts and stromal cells were included, given their known roles in the colorectal cancer microenvironment, CD8^+^ T-cells were included as the primary adaptive immune effector population, and remaining lymphocyte and myeloid subsets were aggregated into composite terms to account for overall immune composition to ensure statistical independence amongst covariates and reduce the influence of overly rare cell-types (i.e., retain degrees of freedom). Further, multicollinearity among covariates was assessed using variance inflation factors (VIF; Supplemental Table S6). Given the modest sample size and exploratory nature of this study, loci with p < 0.001 were considered significant for inclusion in downstream analysis. Lymph-node and distant metastases may arise through distinct biological mechanisms rather than representing sequential stages of progression.^33^ Accordingly, two additional pairwise EWAS contrasts were performed: one comparing non-metastatic tumors to lymph-node–only metastasis, and a second comparing lymph-node–only to distant metastasis. Functional context for genes annotated to significant CpGs was assessed through literature review rather than formal gene set enrichment analysis, given the limited number of loci reaching significance. As a sensitivity analysis, each EWAS contrast was additionally fit using a reduced model excluding all cell-type covariates (age, sex, and MLH1 status only) to assess the contribution of cellular composition adjustment to the identified associations (Supplementary Figure S4).

The genomic distribution of differentially methylated CpG loci was evaluated for depletion or enrichment across annotated genomic contexts (CpG islands, shores, shelves, open sea, and gene promoters). To account for probe-type distribution differences between Infinium Type I (37.4%) and Type II (62.6%) chemistries within the tested CpG set, expected genomic context proportions were weighted by the relative abundance of each probe type. Overall enrichment was assessed using chi-square tests with Monte Carlo simulation (100,000 replicates). Per-category enrichment was assessed using Fisher’s exact test to obtain odds ratios with 95% confidence intervals for each genomic context within each EWAS contrast and methylation direction (Fig. 2C). Enrichment was tested separately for each EWAS contrast (any metastasis, lymph node-only, and distant metastasis). The background for enrichment testing was restricted to the 68,331 CpGs tested in the EWAS after variance filtering, rather than the full EPIC array, to ensure the denominator reflected the actual tested CpG set.

### External Validation

To validate findings, we analyzed Illumina HumanMethylation450K data from TCGA Colon Adenocarcinoma samples, obtained via Bioconductor TCGAbiolinks and processed using the same pipeline as the discovery cohort (SeSAMe normalization, M-value transformation, HiTIMED/EpiDISH cell-type deconvolution). TCGA samples were restricted to pT3 tumors (n = 181) and stratified by identical metastatic criteria (pT3N0M0, pT3N+M0, pT3N±M1). After quality control and removal of sex chromosome probes, 473,864 CpGs were available for analysis. The discovery cohort was profiled on the EPIC array while TCGA used the 450K platform; accordingly, validation was restricted to discovery CpGs with p < 0.001 that were present on both platforms. For each CpG, we tested the matched metastatic contrast using limma linear models with empirical Bayes moderation, adjusted for age, sex, MLH1 status, and cell-type composition. CpGs were considered validated if they showed consistent direction of effect and one-sided significance (p < 0.05) in the matched TCGA contrast.

### Methylation-Expression Correlation Analysis

To assess whether metastasis-associated methylation changes correspond to differences in gene expression, we evaluated correlations between DNA methylation and gene expression using matched TCGA-COAD samples with both 450K methylation and RNA-seq data. Discovery CpGs significantly associated with any metastatic outcome (p < 0.001) were filtered for presence on the 450K platform, yielding CpG-to-gene mappings from the Illumina HumanMethylation450K annotation (IlluminaHumanMethylation450kanno.ilmn12.hg19). CpGs lacking gene annotations and those mapping to genes not quantified in TCGA RNA-seq were excluded. Remaining CpG-gene pairs were carried forward for correlation analysis. Gene expression data (TPM values) were obtained from TCGA RNA-seq and log_2_-transformed after adding a pseudocount of 1. For genes with multiple transcript annotations, expression was averaged. Samples with matched methylation, expression, and clinical data were retained (N = 347). Spearman correlations (ρ) were calculated for each CpG-gene pair, and p-values were adjusted for multiple testing using the Benjamini-Hochberg method. Genomic context for significant correlations was annotated using UCSC RefGene groups (TSS200, TSS1500, 5’UTR, 1st exon, gene body) and relation to CpG islands.

## RESULTS

### Cell Type Metastasis Associations

**Figure 1:**
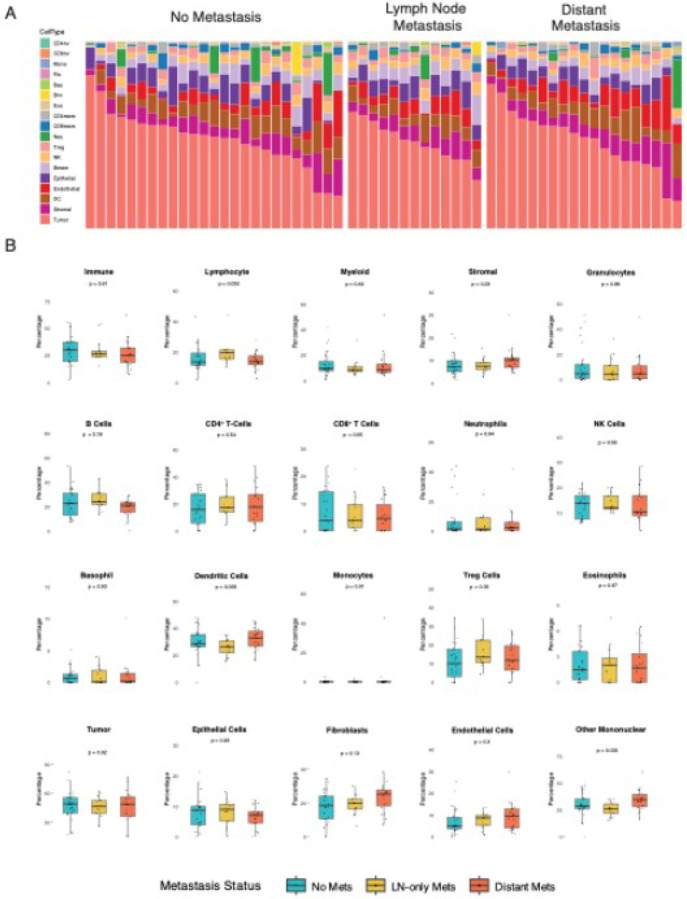
Cell type proportions obtained using Methylation Cytometry Deconvolution

Given that cell-type heterogeneity can confound bulk tissue methylation measurements, we first characterized tumor microenvironment composition across metastatic groups. Figure 1A shows stacked bar charts with cellular composition in each primary tumor from methylation cytometry deconvolution. Epithelial fractions estimated by HiTIMED and EpiDISH were strongly correlated (R = 0.815; Supplementary Figure S3), supporting their combined use in this analysis. Across metastatic severity, tumors show a broadly similar overall profile of cell composition. Across all metastatic categories, tumor cells comprised the largest inferred fraction, with stromal and immune populations representing smaller but variable components (Fig. 1A). Summary statistics for measured cell-type proportions across metastatic groups are provided in Supplemental Table S1. When aggregating cell types into functional compartments, immune, lymphocyte, myeloid, stromal, and granulocyte proportions did not differ significantly across metastatic groups (all p > 0.05; Fig. 1B). Individual cell-type comparisons similarly revealed no statistically significant differences associated with metastatic status for CD4 + T-cells, CD8 + T-cells, B cells, NK cells, Treg cells, dendritic cells, monocytes, neutrophils, basophils, eosinophils, fibroblasts, epithelial cells, endothelial cells, or tumor cell fractions (all p > 0.05; Fig. 1B). Only other mononuclear cells reached nominal significance (p = 0.033), though this finding did not remain significant after multiple testing correction. Tumor samples differed significantly from both non-cancerous reference tissues across the majority of cell types assessed (all p < 0.01; Supplementary Figure S1). CD8^+^ T-cell proportions were reduced in tumor samples relative to both healthy and tumor-adjacent colon, while Treg proportions were elevated in tumor tissue. Fibroblast, epithelial, and endothelial proportions also differed significantly between tissue types.

(A) Stacked bar plots showing estimated proportions of 18 cell types for each patient sample, ordered by metastatic status (No Metastasis, n = 25; Lymph Node Metastasis, n = 13; Distant Metastasis, n = 19). Cell-type proportions were estimated using HiTIMED and EpiDISH. (B) Comparison of aggregated compartments (Immune, Lymphocyte, Myeloid, Stromal, Granulocytes) and individual cell types across metastatic groups. Immune cell proportions are expressed as percentages of the total immune fraction within each sample. Boxplots show median (center line), interquartile range (box), and 1.5 × IQR whiskers. Individual point represent samples. P-values from Kruskal-Wallis tests are shown.

### EWAS

**Figure 2:**
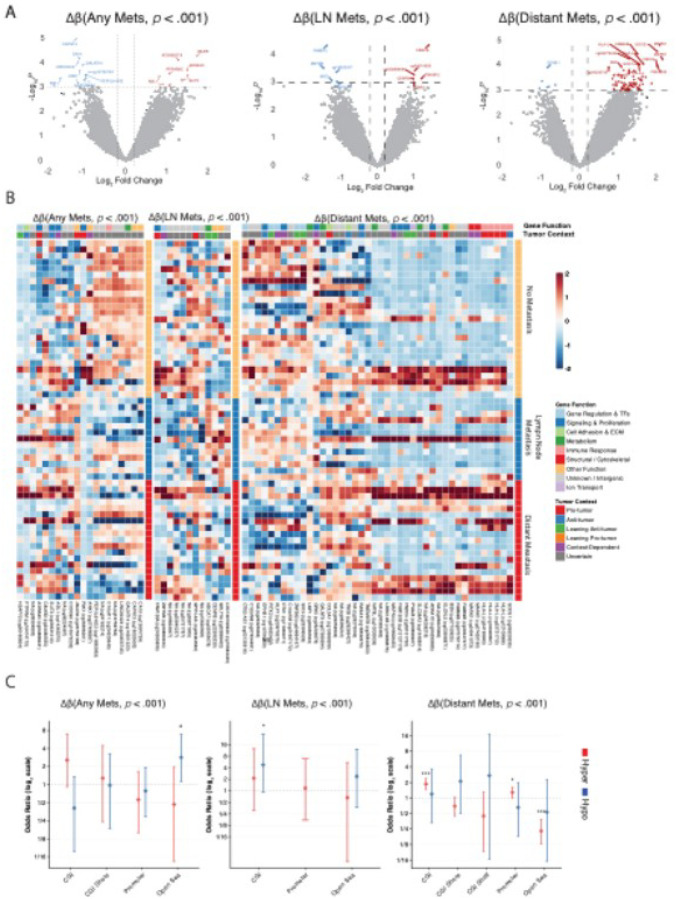
Epigenome-Wide Characterization of Differential DNA Methylation Across Metastatic Phenotypes in Colorectal Cancer

Epigenome-wide association analyses were performed using models adjusted for age, sex, MLH1 status, and cell-type composition (i.e., tumor proportion, fibroblast, stromal, CD8^+^ T-cell, and other lymphocyte fractions, also see Methods). When comparing patients with any metastasis to those without (Fig. 2A, left), we identified 30 CpG sites that were differentially methylated at a significance threshold of p < 0.001 (Table 2; Supplementary Table S2). When comparing non-metastatic tumors to those with lymph node involvement (Fig. 2A, middle), we identified 10 significant CpG sites (p < 0.001; Supplementary Table S3). When comparing non-metastatic tumors to those with distant metastasis (Fig. 2A, right), we identified 174 differentially methylated CpG loci (p < 0.001; Supplementary Table S4), with one CpG shared across metastatic contrasts. When comparing lymph node only to distant metastasis, 164 CpG loci were differentially methylated (p < 0.001; Supplementary Figure S2, Supplementary Table S5). Multicollinearity among covariates was assessed using variance inflation factors (VIF); all covariates had VIF < 3, indicating no problematic collinearity (Supplemental Table S6).

To assess whether the metastasis-associated methylation signal was driven by differences in bulk cellular composition rather than by intrinsic methylation differences, each EWAS contrast was re-run using a reduced model adjusted only for age, sex, and MLH1 status. Effect-size estimates were broadly concordant between the unadjusted and cell-type-adjusted models across all 68,331 tested CpGs (Pearson r = 0.88, 0.78, and 0.89 for any, lymph node–only, and distant metastasis contrasts, respectively; Supplementary Figure S4C). Across all three contrasts, the majority of cell-type-adjusted hits were not detected in the unadjusted analysis (any metastasis: 24/30, 80.0%; lymph node–only: 10/12, 76.9%; distant metastasis: 173/174, 99.4%; Supplementary Figure S4B), indicating that cell-type adjustment sharpened detection of metastasis-associated signal rather than eroding it. This effect was most pronounced for distant metastasis, where only a single CpG (*SOCS1*) reached significance in the unadjusted model compared with 174 after adjustment (Supplementary Figure S4A; compare Fig. 2A).

### Genomic Distribution of Differentially Methylated Loci

To characterize the genomic distribution of metastasis-associated DMPs, we assessed enrichment of significant CpGs (p < 0.001) across CpG islands, shores, shelves, open sea, and promoter regions relative to all CpGs tested in the EWAS (n = 68,331). In the distant metastasis contrast, hypermethylated DMPs were significantly enriched in CpG islands (OR = 1.86) and promoter regions (OR = 1.28), and depleted from open sea regions (OR = 0.23). In the any-metastasis contrast, hypomethylated DMPs were significantly enriched in open sea regions (OR = 2.84). In the lymph node only contrast, hypomethylated DMPs were significantly enriched in CpG islands (OR = 4.75) and depleted from promoter regions (Fisher’s exact test, p < 0.05 for all reported ORs; Fig. 2C; Supplementary Figure S6).

### Gene level annotation of differentially methylated loci

Genes annotated to differentially methylated loci (p < .001) were examined for known functional roles in colorectal cancer based on published literature (Fig. 2B; Table 2). Hypomethylated loci mapped to genes implicated in Wnt/β-catenin and PI3K/Akt signaling, transcriptional regulation, calcium signaling, apoptosis, and epithelial–mesenchymal transition, including *PAK1, ADAMTS18, CREB5, CACNA1C, ANK1*, and *CRYAB*.^34–42^ Hypermethylated loci mapped to genes involved in Wnt signaling, transcriptional regulation, cytoskeletal structure, cell adhesion, immune regulation, and intracellular signaling, including *RNF6, FZD10, TCF12*, *SPTBN4, PTPRM, SOCS1*, and *PIK3R6*.^43^ Effect sizes and p-values for representative CpG loci are reported in Table 2, while Fig. 2B summarizes pathway-level methylation patterns across metastatic categories.

(A) Volcano plots showing differentially methylated positions (DMPs, p < 0.001) comparing any metastasis (left), lymph node-only metastasis (center), or distant metastasis (right) to non-metastatic tumors. Dashed lines indicate p = 0.001 (horizontal) and effect size thresholds (vertical). Blue: hypomethylated; red: hypermethylated in metastatic samples. (B) Heatmap of significant DMPs (p < 0.001) showing Z-score normalized methylation β-values. Columns grouped by EWAS contrast (any, lymph node-only, distant metastasis); rows ordered by metastatic status. Gene annotations and functional pathways shown at top. (C) Odds ratios for genomic context enrichment of hypermethylated and hypomethylated DMPs across the three metastasis contrasts, relative to all CpGs tested in the EWAS (n = 68,331). Odds ratios are plotted on a log_2_ scale; dashed line indicates OR = 1 (no enrichment). Error bars show 95% confidence intervals from Fisher’s exact test. Genomic contexts with zero DMPs in a given contrast are omitted. The LN Only hypomethylated CGI enrichment (OR = 4.75, p = 0.032) has a 95% CI that includes 1.0 (0.92–30.6), reflecting the small number of DMPs (n = 8) in this contrast. *p < 0.05, ***p < 0.001.

### Validation of EWAS Findings in External Cohort

Of the CpGs reaching epigenome-wide significance in the discovery cohort, two were present on the 450K platform, showed consistent direction of effect, and reached one-sided significance in matched TCGA contrasts: *ANK1* (cg27498387, any-metastasis contrast, logFC = − 0.47, p = 0.042) and a DNase I hypersensitivity site (cg09390241, distant metastasis contrast, logFC = 0.63, p = 0.029).

Spearman correlations between DNA methylation (β-value) and gene expression (log_2_ TPM + 1) for all CpG-gene pairs reaching significance (FDR < 0.05) among metastasis-associated loci identified in the discovery cohort (N = 347 TCGA-COAD samples with matched methylation and RNA-seq data). Bars indicate Spearman ρ for each CpG-gene pair, colored by direction of correlation (blue, negative; red, positive).

### Methylation-Expression Correlations

**Figure 3:**
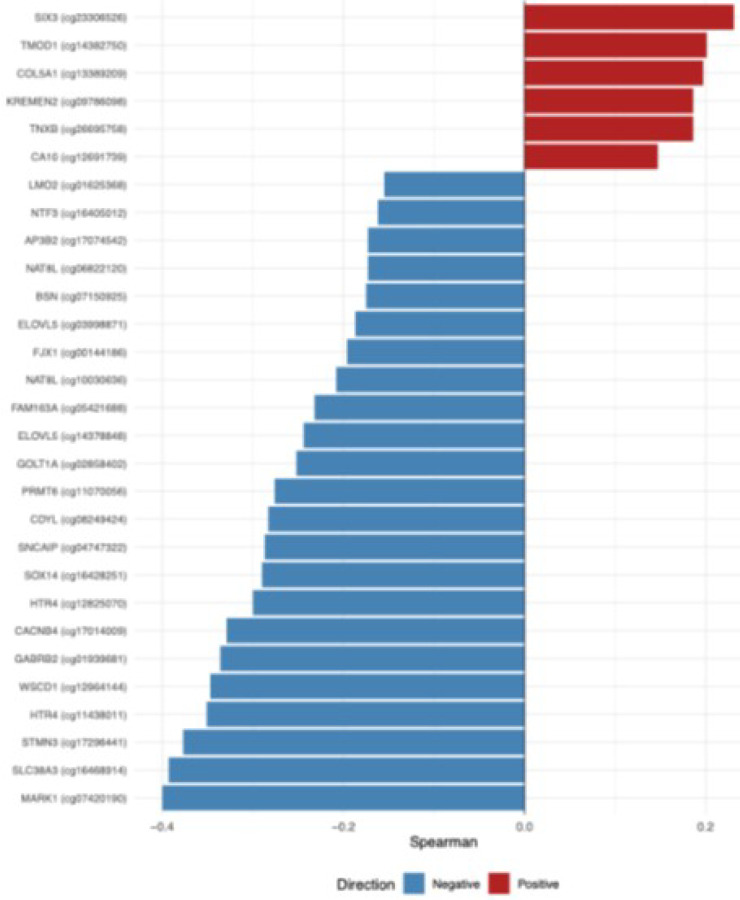
Methylation-expression correlations for metastasis-associated CpGs in TCGA-COAD

To assess whether metastasis-associated methylation changes correspond to differences in gene expression, we evaluated correlations between DNA methylation and RNA expression in TCGA-COAD samples with matched data (N = 347). Of 215 CpGs significantly associated with any metastatic outcome in the discovery cohort, 162 (75%) were present on the 450K platform. After excluding 33 intergenic CpGs and 35 mapping to genes not quantified in RNA-seq, 94 CpG-gene pairs were tested for methylation-expression correlation. Among these, 29 CpG-gene pairs showed significant correlations after multiple testing correction (FDR < 0.05), with 23 exhibiting negative correlations between methylation and expression (Table 3). The strongest negative correlations included CpGs mapping to *MARK1* (ρ = −0.40), *SLC38A3* (ρ = −0.39), and *STMN3* (ρ = −0.38), genes involved in microtubule dynamics, amino acid transport, and cytoskeletal regulation, respectively (Fig. 3; Supplementary Figure S5). Negatively correlated CpG-gene pairs were enriched in promoter-proximal regions compared to all tested CpGs (82.6% vs 59.2%, Fisher’s exact p < 0.05, OR = 3.24). The majority also resided within CpG islands (87%), though this did not differ significantly from the background rate among tested loci (81.7%, p > 0.05), consistent with the high CpG island representation on the 450K array. Six CpG-gene pairs showed significant positive correlations (Fig. 3; Supplementary Table S7), potentially reflecting gene body methylation associated with active transcription.

## DISCUSSION

Tumor metastasis is a major contributor to poor clinical outcomes for CRC, yet epigenetic determinants of metastatic progression remain poorly understood. We characterized DNA methylation patterns associated with metastasis in 57 pT3 colorectal adenocarcinomas while controlling for tumor microenvironment cellular composition. Metastatic status was not associated with systematic TME cellular compositional differences. Epigenome-wide analyses identified distinct methylation alterations, most prominently in the distant metastasis contrast where 174 CpGs reached significance (p < .001) after cell-type adjustment. These loci converged on several key biological programs, including coordinated silencing of Wnt pathway regulators (*SLIT2*, *FZD10*, *RNF6*, *SOX15*), attenuation of interferon signaling and immune surveillance (*SOCS1*, *ISG15*, *SECTM1*), and disruption of cell adhesion and cytoskeletal integrity (*PTPRM*, *COL5A1*, *ANK1*), with two candidate CpGs showing consistent effects in an independent TCGA-COAD cohort and 23 CpG-gene pairs demonstrating significant methylation-expression correlations.

To interpret metastasis-associated DNA methylation changes in the context of tumor heterogeneity, we first examined whether metastatic progression in this cohort was accompanied by systematic shifts in immune or stromal cell composition. Using methylation cytometry deconvolution, we observed substantial variability in immune and stromal cell proportions across tumors, consistent with prior reports describing heterogeneous immune infiltration patterns in colorectal cancer.^44^ However, we did not observe consistent trends in the abundance of major immune or stromal compartments, including CD8^+^ T-cells, across metastatic categories within this pT3 cohort.^45,46^ In contrast, comparison with non-cancerous reference tissues confirmed that the deconvolution approach captured expected differences in cell-type composition: tumor samples showed reduced CD8^+^ T-cell proportions and elevated Treg fractions relative to both healthy colon and tumor-adjacent tissue (Supplementary Figure S1), consistent with the well-documented immune remodeling that accompanies colorectal tumorigenesis. The absence of significant within-cohort differences across metastatic groups may in part reflect architectural heterogeneity in bulk tissue specimens, as additional measures to control for tumor purity and tissue composition (e.g., macrodissection or purity thresholds) were not applied. However, the clear separation of tumor from non-cancerous reference tissues supports the sensitivity of the deconvolution approach.

Metastatic progression in colorectal cancer was associated with widespread changes in DNA methylation indicative of altered epigenetic states within the tumor microenvironment. Our EWAS identified a progressive reduction in hypomethylated CpG loci with advancing disease severity. This pattern is consistent with global methylation dysregulation previously implicated in genomic instability and metastatic potential.^47^ Differentially methylated loci were mapped to metabolic, gene regulatory, and adhesion-related pathways, highlighting epigenomic remodeling beyond genetic mutation.^48^ The increased burden of methylation changes observed in distant metastases suggests additional epigenetic reprogramming that may reflect adaptation to distinct microenvironmental pressures.^5^ Clinically, these metastasis-specific methylation profiles may improve prognostic accuracy and inform risk stratification.

In our cohort, differentially methylated loci associated with tumor metastasis were observed and persisted after adjustment for cell-type composition (Supplementary Figure S4). These methylation changes demonstrated contrast-specific regional organization across the genome. In the distant metastasis contrast, hypermethylated loci were enriched in CpG islands (OR = 1.86) and promoter regions (OR = 1.28), and depleted from open sea regions (OR = 0.23), consistent with promoter-associated gene silencing as a feature of advanced disease progression.^49^ Hypomethylated DMPs in the any-metastasis contrast were enriched in open sea regions (OR = 2.84), and in the lymph node–only contrast were enriched in CpG islands (OR = 4.75). CpG shores and shelves did not show significant enrichment in any contrast.

Furthermore, the identified loci mapped to functionally important, progression-related gene sets. Hypermethylated loci in metastatic tumors were mapped to genes annotated to Wnt signaling, suggesting epigenetic suppression of regulatory nodes involved in epithelial signaling, chemokine responsiveness, and stromal communication.^50–56^ In addition, hypermethylation of genes associated with cytoskeletal structure and cell adhesion is consistent with disruption of cell–cell interactions and tissue organization during metastatic progression.^57^ Several immune-regulatory genes, including *SOCS1*, *ISG15*, and *SECTM1*, also exhibited hypermethylation, consistent with attenuation of interferon signaling and cytokine feedback control within the tumor microenvironment.^43^ *SOCS1* functions as both a tumor suppressor and a negative regulator of antitumor CD8^+^ T-cell responses; its epigenetic silencing via promoter hypermethylation in metastatic tumors may therefore simultaneously increase tumor aggressivity and impair immune surveillance.^58^ In aggregate, these patterns support a model in which promoter-associated hypermethylation contributes to suppression of epithelial integrity and immune signaling pathways in metastatic colorectal cancer.

Hypomethylated loci in metastatic tumors were mapped to genes involved in epithelial–mesenchymal transition, cytoskeletal remodeling, cell adhesion, and intracellular signaling pathways, suggesting activation of transcriptional programs that support cellular plasticity and invasion.^59^ Hypomethylation of genes associated with calcium signaling and apoptotic regulation further indicates remodeling of stress-response and survival pathways during metastatic progression.^43,55,60–62^ In addition, hypomethylation of immune-related genes and GPCR-associated mediators points to epigenetic modulation of tumor–microenvironment communication, potentially influencing immune signaling and stromal interactions.^63^ Taken together, these patterns are consistent with epigenetic activation of pathways that facilitate invasion, adaptation, and immune modulation in metastatic colorectal cancer.

Examination of candidate CpGs in the independent TCGA-COAD cohort provided partial support for their association with metastatic progression. cg27498387, located in the promoter of the tumor suppressor *ANK1*, was hypomethylated in metastatic samples in both cohorts. Although *ANK1* has been described as a tumor suppressor in some contexts, recent proteomic profiling has identified *ANK1* as a component of the EGFR-centered interactome specifically in metastatic colorectal tumors, suggesting it may contribute to oncogenic signaling and metastatic progression.^64^ cg09390241, situated within an intergenic region overlapping a DNase I hypersensitivity site, likely resides in an active enhancer whose hypermethylation in metastatic tumors could repress nearby tumor-suppressive elements.^65^

Beyond replication of individual CpG sites, we examined whether metastasis-associated methylation changes corresponded to differences in gene expression by assessing methylation-expression correlations in TCGA-COAD. Among 94 testable CpG-gene pairs, 23 exhibited significant negative correlations between promoter methylation and transcript abundance. This pattern is consistent with promoter hypermethylation contributing to transcriptional downregulation.^66,67^ Negatively correlated CpG-gene pairs were enriched in promoter-proximal regions compared to all tested CpGs, consistent with canonical promoter-associated silencing. The strongest correlations involved genes regulating microtubule dynamics (*MARK1, STMN3*), amino acid transport (*SLC38A3*), and histone-mediated transcriptional repression (*CDYL*, *PRMT6*), suggesting that metastasis-associated hypermethylation may silence genes involved in maintaining cellular architecture and chromatin state.^68–72^

Several limitations should be acknowledged. The modest sample size (N = 57) may limit power to detect subtle methylation differences and precludes assessment of effect modification by factors such as tumor sidedness, mismatch repair status, or oncogenic driver mutations (*BRAF/KRAS*). Restriction to pT3 tumors controls for T-stage confounding but may limit generalizability to other tumor stages. Critically, our cross-sectional design cannot establish temporal precedence—the observed methylation patterns could represent either pre-existing drivers of metastasis or secondary consequences of systemic disease, including immune exhaustion or stromal remodeling induced by metastatic spread. Heterogeneity in treatment exposure, tumor histology, and FFPE block age may introduce unmeasured confounding. Longitudinal studies of precancerous lesions and future multi-institutional cohorts with larger, more heterogeneous populations would help distinguish driver from passenger methylation events and improve generalizability. Genomic context enrichment estimates for the any-metastasis and lymph node–only contrasts should be interpreted cautiously given the small number of DMPs (n = 7–24), which limits statistical power for enrichment testing. Future work should include sufficient follow up to study longitudinal outcomes (e.g., local/metastatic recurrence) in the presence/absence of associated high risk factors. While our analyses identified methylation changes associated with metastasis and corresponding expression differences, the cell-type specificity of these alterations remains unresolved. Tissue sections were not subjected to macro-dissection to isolate tumor epithelium, as we sought to capture methylation signals from the broader tumor microenvironment including immune and stromal compartments. However, this approach means that observed methylation differences may reflect contributions from multiple cell populations rather than tumor-intrinsic changes alone. Integration with single-cell RNA-seq or spatial transcriptomics would help localize these epigenetic changes to specific tumor or microenvironment compartments and distinguish tumor-intrinsic from microenvironment-driven methylation alterations.

## CONCLUSIONS

Colorectal cancer metastasis is driven by coordinated cellular and epigenetic remodeling within the tumor microenvironment. Using methylation cytometry deconvolution and epigenome-wide association analyses, we identified distinct DNA methylation patterns associated with metastatic progression in pT3 colorectal cancer, highlighting coordinated epigenetic remodeling associated with disease progression. These findings provide exploratory evidence nominating candidate methylation-based biomarkers and pathways linked to metastatic progression, motivating larger-scale validation efforts and integration of spatial and single-cell approaches to better understand how epigenetic states shape tumor–immune interactions during colorectal cancer progression.

## Supplementary Material

Supplementary Files

This is a list of supplementary files associated with this preprint. Click to download.

- SupplementalTextandfigures.docx
- SupplimentaryTableS2.csv
- SupplimentaryTableS3.csv
- SupplimentaryTableS4.csv
- SupplementaryTableS5.csv

## Figures and Tables

**Table 1:** Patient Disease Characteristics Stratified by Metastasis

Age (years)	Total (N = 57)	No Nodal or Distant Metastasis (N = 25)	Nodal or Distant Metastasis (N = 32)
	66 (33–93)	68 (42–93)	64 (33–90)
**Female (%)**	24 (42)	11 (44)	13 (41)
**Tumor Grade (%)**			
Well Differentiated (Grade 1)	35 (61)	15 (60)	20 (62)
Moderately Differentiated (Grade 2)	12 (21)	6 (24)	6 (18)
Poorly Differentiated (Grade 3)	10 (17)	4 (16)	6 (18)
**Lymph Node Status (%)**			
N0	44 (77)	25 (100)	19 (59)
N+	13 (22)	0 (0)	13 (40)
**Distant Metastasis Status (%)**			
M0	38 (66)	25 (100)	13 (40)
M1	19 (33)	0 (0)	19 (59)
**Site in Colon (%)**			
Ascending	26 (45)	12 (48)	14 (44)
Transverse	6 (10)	3 (12)	3 (9)
Descending	25 (43)	10 (40)	15 (47)
**MLH1 Status (%)**			
MLH1 Deficient	16 (28)	6 (24)	10 (31)
MLH1 Intact	41 (71)	19 (76)	22 (68)

Note: Nodal or Distant Metastasis includes both lymph node-only metastasis (Stage III, n = 13) and distant metastasis (Stage IV, n = 19). All downstream analyses stratified these groups separately.

**Table 2:** Representative Hyper- and Hypomethylated CpGs Associated with Lymph Node or Distant Metastasis. *Δβ*(*AnyMetastasisp* < 0.001)

CpG_ID	Gene	logFC	P-Value	Genomic Location	Relation to Island
Hyper Methylated					
cg04888241	*ADRA2C*	1.478	2.05E-04	3’UTR, 1stExon	Island
cg06224587	*PPP2R2C*	1.053	2.93E-04	5’UTR	Open Sea
cg26750188	*JMJD8*	1.714	5.43E-05	TSS1500	Island
cg06631010	*SLIT2*	1.494	3.38E-04	TSS1500	Island
cg10573232	*CCDC60*	1.426	4.88E-04	5’UTR TSS1500	Island
cg16474170	*PTPRM*	1.354	4.98E-04	TSS1500	Island
cg26166804	*H2AFY2*	1.359	5.60E-04	1stExon TSS200	Island
cg24480555	*C8orf33*	0.884	7.85E-04	TSS1500	North Shore
cg20964064	*KCNQ2*	1.064	7.99E-04	Body	Island
cg09304803		1.269	7.92E-05	TSS1500;1stExon;5’UTR	Island
cg10655471	*SPTBN4*	1.169	9.94E-04	Body	Island
cg07702509	*SNORA39*	1.053	9.17E-04	TSS200	South Shore
cg13831596	*ATP7B*	0.814	8.23E-04	Body	Open Sea
Hypo Methylated					
cg16756764		−0.884	2.53E-04		Open Sea
cg19221542		−0.885	6.21E-04		North Shore
cg10213101		−0.889	9.21E-04		Open Sea
cg11895993	*FER1L6-AS2*	−0.948	3.25E-04	Exon Band	Open Sea
cg24529549	*C10orf11*	−0.955	4.47E-04	Body	Open Sea
cg04165214		−0.975	3.24E-04		Open Sea
cg24396358	*HLA-DOA*	−1.006	5.21E-04	TSS200	South Shore
cg14501323	*GALNT14*	−1.045	1.30E-04	Body	Open Sea
cg27498387	*ANK1*	−1.110	2.21E-04	TSS1500	Island
cg12691739	*CA10*	−1.121	7.01E-05	Body	Open Sea
cg18886245	*TFAP2B*	−1.151	6.86E-04	Body	South Shore
cg23355745	*LINC00424*	−1.152	9.55E-05	Body	Open Sea
cg21056723	*LOC644145*	−1.180	5.45E-04	TSS200	Open Sea
cg19020602	*CAPN13*	−1.200	1.09E-05	Exon Band	Open Sea
cg26996201	*PAK1*	−1.574	4.40E-04	5’UTR TSS1500	Island
cg11287055	*DSCR3*	−1.724	9.13E-04	Body	Island
cg14362370	*ABL1*	0.810	3.61E-04	5’UTR TSS1500	Island

**Table 3:** Methylation-expression associations for genes annotated to metastasis-associated CpGs in TCGA-COAD

CpG	Gene	Spearman	FDR	Region	Island Context
cg07420190	*MARK1*	−0.4	2.24E-10	Gene Body	CpG Island
cg16468914	*SLC38A3*	−0.393	2.79E-10	Promoter-proximal (5’UTR/1stExon)	CpG Island
cg17296441	*STMN3*	−0.377	1.64E-09	Gene Body	CpG Island
cg11438011	*HTR4*	−0.351	2.82E-08	Promoter (TSS)	CpG Island
cg12964144	*WSCD1*	−0.347	3.54E-08	Promoter-proximal (5’UTR/1stExon)	CpG Island
cg01939681	*GABRB2*	−0.336	1.04E-07	Promoter-proximal (5’UTR/1stExon)	CpG Island
cg17014009	*CACNB4*	−0.329	1.74E-07	Gene Body	CpG Island
cg12825070	*HTR4*	−0.3	2.93E-06	Promoter-proximal (5’UTR/1stExon)	CpG Island
cg16428251	*SOX14*	−0.29	7.02E-06	Promoter (TSS)	CpG Island
cg04747322	*SNCAIP*	−0.287	8.43E-06	Promoter (TSS)	CpG Shore
cg08249424	*CDYL*	−0.283	1.03E-05	Promoter (TSS)	CpG Island
cg11070056	*PRMT6*	−0.276	1.79E-05	Promoter-proximal (5’UTR/1stExon)	CpG Island
cg02858402	*GOLT1A*	−0.252	1.24E-04	Promoter (TSS)	Open Sea
cg14378848	*ELOVL5*	−0.244	2.10E-04	Promoter-proximal (5’UTR/1stExon)	CpG Island
cg05421688	*FAM163A*	−0.232	5.01E-04	Promoter (TSS)	CpG Shore
cg10030636	*NAT8L*	−0.208	0.00231999	Promoter (TSS)	CpG Island
cg00144186	*FJX1*	−0.196	0.00422172	Promoter-proximal (5’UTR/1stExon)	CpG Island
cg03998871	*ELOVL5*	−0.187	0.00678511	Promoter-proximal (5’UTR/1stExon)	CpG Island
cg07150925	*BSN*	−0.175	0.01203793	Promoter (TSS)	CpG Island
cg06822120	*NAT8L*	−0.173	0.01278759	Promoter (TSS)	CpG Island
cg17074542	*AP3B2*	−0.173	0.01278759	Promoter (TSS)	CpG Island
cg16405012	*NTF3*	−0.162	0.02145689	Gene Body	CpG Island
cg01625368	*LMO2*	−0.155	0.02977348	Promoter-proximal (5’UTR/1stExon)	CpG Island

## Data Availability

The datasets generated and/or analysed during the current study are available in the Data can be accessed through the Gene Expression Omnibus, [Link will be provided once the paper is accepted]. For reproducibility the source code used to generate the figures and tables have been deposited on Zenodo (doi: 10.5281/zenodo.20014575)

## Abbreviations

CRC: colorectal cancer
TME: tumor microenvironment
DNAm: DNA methylation
TIL: Tumor-infiltrating lymphocytes
CAF: cancer-associated fibroblasts
HiTIMED: Hierarchical Tumor Immune Microenvironment Epigenetic Deconvolution
EpiDISH: Epigenetic Dissection of Intra-Sample Heterogeneity
MSI: microsatellite instability
FFPE: formalin-fixed paraffin-embedded
VIF: Variance Inflation Factor
GTEx: Genotype-Tissue Expression
EWAS: epigenome-wide association study
IQR: interquartile range
DMP: differentially methylated positions
